# Bovine CD2^-^/NKp46^+ ^cells are fully functional natural killer cells with a high activation status

**DOI:** 10.1186/1471-2172-7-10

**Published:** 2006-04-27

**Authors:** Preben Boysen, Ingrid Olsen, Ingvild Berg, Siri Kulberg, Grethe M Johansen, Anne K Storset

**Affiliations:** 1 Department of Food Safety and Infection Biology, Norwegian School of Veterinary Science, P.O.Box 8146 Dep., N-0033 Oslo, Norway; 2 Department of Animal Health, National Veterinary Institute, P.O.Box 8156 Dep., N-0033 Oslo, Norway

## Abstract

**Background:**

Natural killer (NK) cells in the cow have been elusive due to the lack of specific NK cell markers, and various criteria including a CD3^-^/CD2^+ ^phenotype have been used to identify such cells. The recent characterization of the NK-specific NKp46 receptor has allowed a more precise definition of bovine NK cells. NK cells are known as a heterogeneous cell group, and we here report the first functional study of bovine NK cell subsets, based on the expression of CD2.

**Results:**

Bovine CD2^- ^NK cells, a minor subset in blood, proliferated more rapidly in the presence of IL-2, dominating the cultures after a few days. Grown separately with IL-2, CD2^- ^and CD2^+ ^NK cell subsets did not change CD2 expression for at least two weeks. In blood, CD2^- ^NK cells showed a higher expression of CD44 and CD25, consistent with a high activation status. A higher proportion of CD2^- ^NK cells had intracellular interferon-gamma in the cytoplasm in response to IL-2 and IL-12 stimulation, and the CD2^- ^subset secreted more interferon-gamma when cultured separately. Cytotoxic capacity was similar in both subsets, and both carried transcripts for the NK cell receptors KIR, CD16, CD94 and KLRJ. Ligation by one out of two tested anti-CD2 monoclonal antibodies could trigger interferon-gamma production from NK cells, but neither of them could alter cytotoxicity.

**Conclusion:**

These results provide evidence that bovine CD2^- ^as well as CD2^+ ^cells of the NKp46^+ ^phenotype are fully functional NK cells, the CD2^- ^subset showing signs of being more activated in the circulation.

## Background

Natural killer cells were initially defined as a heterogeneous population of large granular lymphocytes that were able to spontaneously kill target cells. Human natural killer cells were later phenotypically defined as CD3^-^/CD56^+ ^lymphocytes [1], and can be divided into distinct functional subsets, the CD56^bright ^being mainly cytokine producers and CD56^dim ^being more effective killers [2]. Mice and rats do not express CD56 on haematopoietic cells; thus NK cells in these species are instead defined as non-T lymphocytes bearing NKR-P1 molecules [3], although the use of NKp46 as the standard NK cell identifier in the rat has been suggested [4]. There is less evidence for a clear functional division of NK cells in rodents [5]. Criteria for the definition of NK cells have not been clarified in most farm animal species [6]. In the cow, NK-like cells have been described as CD3^-^/CD2^+ ^lymphocytes [7-10], and NK-like cells have been isolated on the basis of markers not commonly expressed by NK cells [11]. Recently, the characterization of bovine NKp46 (CD335) enabled a more precise identification of bovine NK cells [12]. The NKp46 receptor has been proposed as the most accurate marker for human NK cells [13], as it is highly NK-restricted and uniformly expressed by cultured as well as resting NK cells [14-16].

The CD2 molecule is known as an adhesion molecule as well as a receptor for activation on both NK cells and T-cells, reviewed in [17]. In the cow, CD2 is frequently used as a cell marker in cellular immunology, and several anti-bovine CD2 monoclonal antibodies (mAbs) have been described [18,19]. The functional role of CD2 in bovine NK cells has not been previously studied, but NKp46^+ ^cells in cattle peripheral blood can be divided into distinct CD2^+ ^and CD2^- ^phenotypes, the latter typically comprising around 20% of the cells [12]. Similar proportions have been reported in human blood [20,21], but only a few studies have been published that functionally describe CD2^- ^NK cells from blood in healthy humans [22-24]. There does not seem to be a strong difference in expression of CD2 between the human CD56^bright ^and CD56^dim ^NK cell subsets [2]. In the mouse, CD2^- ^cells has been associated with immature cell stages [5], but blocking and knock-out experiments have shown that CD2 is not necessary for well-developed NK cell function [25,26]. Accordingly, CD2 has been classified as a co-receptor [27], although the use of this term has been questioned, since apparently no known activating NK cell receptor stand out as dominating, unlike in T-cells [16].

The scope of the present study was to investigate bovine NK cell subsets, defined by CD2 expression. Our results show that both subsets are fully capable of NK cell activity, and blocking experiments confirm a redundancy of CD2 for NK cell function. Furthermore, the CD2^- ^subset is more activated in circulation and show a stronger proliferative and interferon-gamma (IFN-γ) producing capacity.

## Results

### CD2 expression in peripheral blood and IL-2 activated NK cells

**Figure 1:**
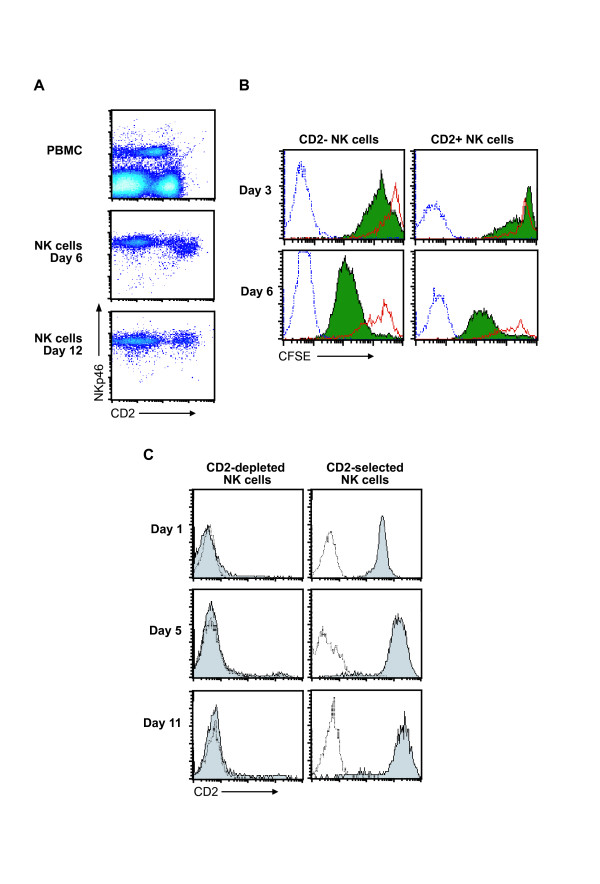
**CD2 distribution on bovine NK cells in peripheral blood and during cell culture**. (A) CD2 expression by PBMCs from healthy cattle, and in NKp46-selected cells cultured for the indicated time in the presence of 100U/ml rbIL-2. Results were obtained by two-colour flow cytometry, gating for viable lymphocytes. Data shown are from one animal representative of 19. (B) Monitoring of cell divisions of CD2^- ^and CD2^+ ^NK cell populations during three and six days of rbIL-2 (100U/ml) stimulation. NKp46-selected cells were labelled with CFSE the day after isolation and CFSE intensity (filled histograms) measured in flow cytometry on the indicated days, gating for CD2^- ^or CD2^+ ^viable cells. Solid line open histograms show non-dividing NK cells, cultured in the presence of 1U/ml rbIL-2, while broken line open histograms show unlabeled stimulated NK cells. Data shown are from one animal representative of five. (C) Stability of CD2 expression in NK cell subsets cultured separately. NK cells separated with immunomagnetic beads for absence or presence of CD2 were cultured separately for eleven days in the presence of IL-2, monitoring CD2 expression at three different time points (filled histograms). Day 1 corresponds to the day of separation, 24 h after primary NK cell isolation. Open histograms show secondary mAb controls. Data shown are from one animal representative of eight.

Expression of CD2 was analyzed on NK cells from fresh peripheral cattle blood, and on isolated NK cells followed for eleven days in the presence of recombinant bovine interleukin-2 (rbIL-2) (Fig. 1A). In peripheral blood, NKp46^+ ^cells were divided into a major CD2 expressing subset and a minor CD2 non-expressing subset, the latter typically comprising between 15 to 30% of the NK cells. Following rbIL-2 activation, a moderate increase in the CD2 intensity was observed for both subsets, thus the initially negative population will in the following be named CD2^-/low ^when rbIL-2 activated. The proportion of CD2^-/low ^cells increased during eleven days of culture and comprised 60–90% of the cells at the end of the period; this pattern was maintained for at least three weeks (not shown).

To elucidate the proliferation of these subsets, NK cells were stained with the cell division marker carboxyfluorescein diacetate succinimidyl ester (CFSE) on the day after isolation, and cultured in the presence of rbIL-2, using quiescent NK cells as controls [28]. Three days later, the CD2^-/low ^subset had decreased more in CFSE intensity than the CD2^+ ^subset, indicating that the former subset had proliferated more vigorously (Fig. 1B). However, after six days in culture, CFSE intensity in the two subsets were almost similar, indicating that CD2^+ ^NK cells entered proliferation at a later stage than the CD2^-/low ^cells.

To further clarify if the CD2^+ ^and CD2^-/low ^cells were distinct subsets, NK cells were divided by immunomagnetic beads into CD2 expressing and non-expressing subsets the day after NK cell isolation, and cultured in rbIL-2 containing medium for ten consecutive days. Throughout this period, no major change of the expression of CD2 was observed (Fig. 1C). The tendency of a low CD2 expression on part of the CD2^- ^subset, as seen with non-separated cells, was not clear in these separated cell cultures. A frequent observation made was that the CD2-depleted NK cells entered an exponential growth phase at an earlier stage than the CD2 selected cells, in accordance with the CFSE labelling results.

### Phenotype of CD2-defined subsets of NK cells in peripheral blood and following IL-2 activation

**Figure 2:**
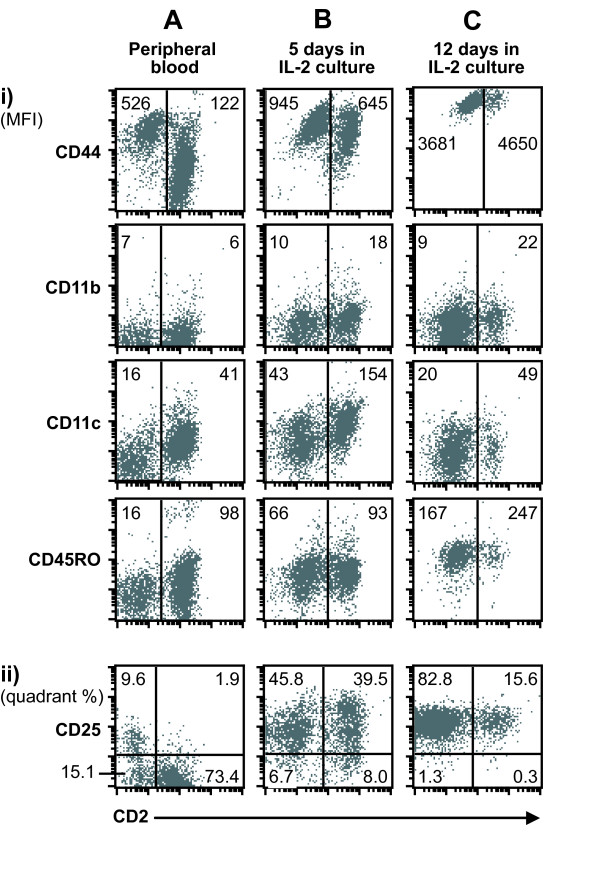
**Expression of activation markers by CD2^+ ^and CD2^- ^subsets of bovine NK cells**. Scatter plots show NKp46 expressing lymphocytes, with CD2 in the x-axis and the indicated marker in the y-axis. PBMCs were analyzed in peripheral blood (A), and isolated NKp46^+ ^cells of the same sample was cultured in the presence of rbIL-2 and analyzed again after 5 (B) and 12 (C) days. Fluorescence settings in the flow cytometer were kept unchanged and verified using secondary controls (not shown). Mean fluorescence intensity (MFI) of the y-axis for each subset is shown for markers showing a continous distribution (i), while quadrant percentages are shown for one marker with a biphasic distribution (ii). Data shown are from one animal representative of eight.

The expression of selected surface markers in relation to CD2 expression on NK-cells was analyzed in freshly drawn peripheral blood, and following culture of isolated NK cells for 5 and 12 days. In peripheral blood, the greatest difference was seen in expression of CD44, which was brightly expressed by all CD2^- ^NK cells, and present but more spread out at lower intensities on CD2^+ ^NK cells (Fig. 2A). Most NK cells were weakly CD11c positive, but for this marker the CD2^- ^subset labelled less intensely than the CD2^+ ^subset. Similarly, CD45RO was less intensely expressed by the CD2^- ^subset. CD11b was expressed by a minor part of NK cells, similarly in both subsets. A minor part of NK cells, but proportionally more within the CD2^- ^subset, expressed CD25 (IL-2Rα).

Following isolation and culture of bovine NK cells for five days in the presence of rbIL-2, differences between the subsets became less clear (Fig. 2B). At this time CD44 and CD25 were clearly upregulated and expressed by almost all NK cells, CD11c and CD45RO were slightly upregulated while CD11b remained unchanged. CD44 remained clearly brighter on the CD2^-/low ^subset and CD11c slightly brighter on the CD2^+ ^subset.

Following 12 days in rbIL-2 culture, a further upregulation in both subsets was seen for CD44 (intensely bright), CD45RO and CD25, while CD11c had decreased, and CD11b remained unchanged (Fig. 2C). At this time, differences between the CD2^-/low ^and CD2^+ ^subsets were not readily distinguishable. After three weeks, as primary NK cell cultures started to show reduced viability, a similar phenotype pattern was seen, although CD44 and CD45RO expression was even further increased in all NK cells (not shown).

### Evaluation of mRNA expression of NK receptor genes in CD2^- ^and CD2^+ ^NK cell subsets

**Figure 3:**
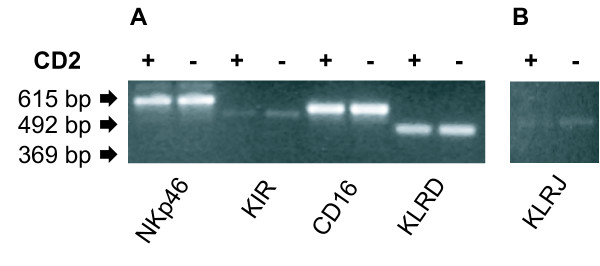
**RT-PCR analysis of NK cell receptor genes in CD2^+ ^and CD2^- ^NK cell subsets**. (A) Separated NK cell subsets were cultured for one week in the presence of rbIL-2 and analyzed for the indicated NK cell receptors at the mRNA transcript level. Results show products following 25 PCR cycles, from one animal representative of four. (B) The KLRJ gene analyzed as for panel A, following 30 PCR cycles.

mRNA was extracted from NK cell subsets selected as described above and cultured for one week in the presence of rbIL-2. Standardized amounts of cDNA synthesized from these transcripts were analyzed for gene expression using RT-PCR. Analysis at 25 cycles showed that the two NK cell subsets expressed the NK receptor genes NKp46 (CD335), KIR, FcγRIIIA (CD16), KLRD1 (CD94) (Fig. 3A) [29,30]. No difference between the subsets measured as intensity of bands in agarose gel was detected. The KLRJ gene was considerably less expressed than the other receptors, and only appeared following 30 cycles (Fig. 3B). These results demonstrate that common NK cell receptors are expressed by both the CD2^-/low ^and CD2^+ ^subsets, confirming the NK nature of these cells.

### IFN-γ production by CD2^- ^and CD2^+ ^NK cell subsets in peripheral blood and culture

**Figure 4:**
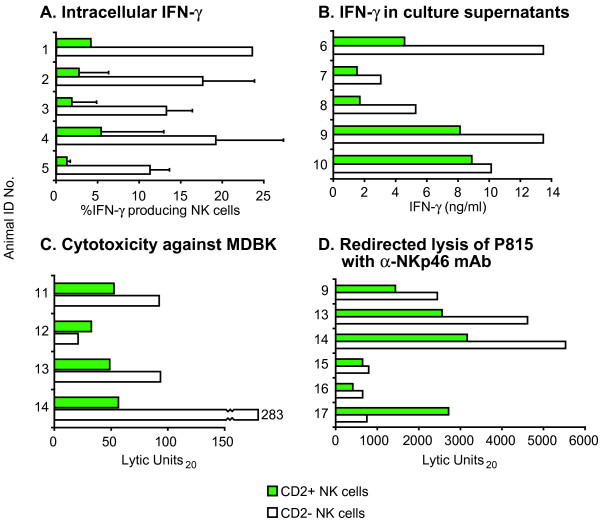
**Functional properties of CD2^- ^and CD2^+ ^bovine NK cell subsets**. (A) Intracellular IFN-γ response in bovine heparinized whole blood, stimulated for18 hrs with 200U/ml rbIL-2 and 5U/ml rbIL-12. Cells were surface labelled and gated for NKp46^+ ^and CD2^+ ^or CD2^-^, and bars represent net per cent cells positive for cytoplasmatic IFN-γ (stimulated samples minus unstimulated controls). Each of five tested animals is shown individually; error bars represent SD of repeated assays (*n *= 1–3). (B) IFN-γ measured in supernatants of bovine NK cells separated with respect to CD2 expression, cultured for around one week in the presence of 100U/ml rbIL-2 and stimulated for the last 24 hours with 0.5U/ml rbIL-12. Responses were measured by an ELISA and are shown as stimulated samples minus unstimulated controls for individual animals. (C) Cytotoxicity of NK cell subsets obtained likewise, against the bovine kidney cell line MDBK, in a 4 hrs ^51^Cr release assay. Bars represent four individual animals and show lytic units (LU) defined as stated in *Methods*. (D) Redirected lysis of FcR-bearing murine P815 target cells by similar NK cell subsets, in the presence of α-NKp46 mAb, calculated and presented as in (C).

To assess the capacity for IFN-γ production by peripheral blood NK cells, heparinized whole blood was cultured overnight in the absence or presence of rbIL-2 and rbIL-12 and analyzed in flow cytometry for intracellular IFN-γ in relation to NKp46 and CD2 surface expression. Non-stimulated cultures contained very few IFN-γ positive cells, and these numbers were used as baseline levels to calculate net increase in stimulated cultures. From cultures stimulated by both rbIL-2 and rbIL-12, the CD2^-/low ^NK cells had a markedly higher proportion of IFN-γ producing cells than the CD2^+ ^subset (Fig. 4A).

IFN-γ secretion by rbIL-2 activated NK cell subsets was assessed by separating NK cells into CD2^- ^and CD2^+ ^subsets and cultured for one week as described above. Following 24 hrs further incubation in the absence or presence of rbIL-12, the level of IFN-γ in the supernatant was measured in an ELISA. In the presence of rbIL-12, the CD2^-/low ^subset produced from 10 to 300% more IFN-γ than CD2^+ ^cells (Fig. 4B). IFN-γ production in the absence of rbIL-12 was negligible (< 1 ng/ml) by both subsets, and were subtracted as baseline levels in the calculation of these responses. The expression of CD2 was unchanged during this period, as shown above.

### Cytotoxicity of CD2^- ^and CD2^+ ^NK cell subsets

**Figure 5:**
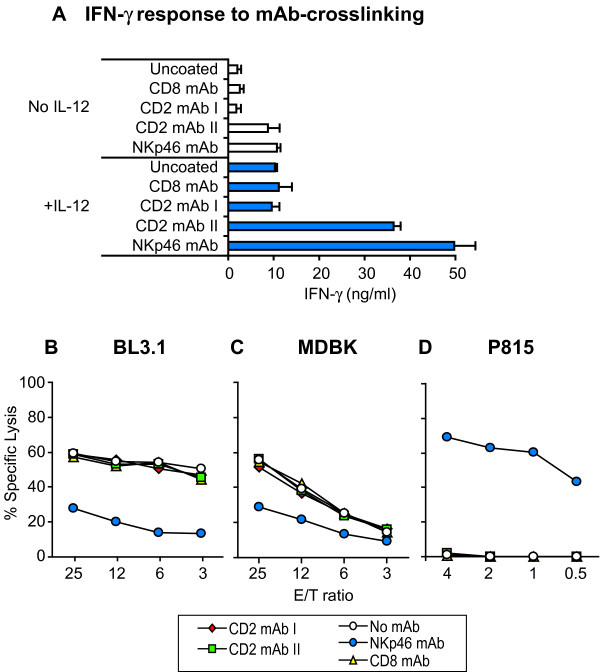
**Effect of anti-CD2 mAbs on bovine NK cell functions**. (A) IFN-γ response from NK cells caused by cross-linking of CD2. Plastic wells were pre-coated with two different mAbs against bovine CD2, along with NKp46-mAb as positive control and CD8-mAb or medium as negative controls. rbIL-2 activated NK cells were added and incubated for 24 h in the presence or absence of rbIL-12 (0.5 U/ml), and IFN-γ content in the supernatants analyzed by an ELISA. Data shown are means ± SD of two to four individual animals. (B-C) IL-2 activated NK cell killing of the bovine target lines BL3.1 and MDBK in a 4 h ^51^Cr-release assay, with addition of the indicated mAbs for blocking. CD2mAb I = BAQ95A, CD2 mAb II = MUC2A, CD8 mAb = CACT80C, NKp46 mAb = AKS1. (D) As above, using murine Fc-receptor bearing P815 target cells to obtain a redirected lysis effect. Data shown in (B-D) are from one animal representative of three to five.

CD2^+ ^and CD2^-/low ^NK cell subsets, isolated and cultured separately for about one week in the presence of rbIL-2, were analyzed for cytotoxicity in a ^51^Cr release assay, using two previously described target cell lines [12]. The bovine MDBK cell line was killed moderately more efficiently by the CD2^-/low ^subset compared with the CD2^+ ^subset by three out of four animals tested (Fig. 4C). Addition of anti-NKp46 mAb partly inhibited killing by both subsets in a similar fashion as previously reported [12] and shown below (Fig. 5C). In a redirected lysis assay using anti-NKp46 mAb and the murine FcR-bearing targets P815, the CD2^-/low ^NK cells was clearly the more cytotoxic subset in three of six animals tested, almost equal in two, and less cytotoxic in one (Fig. 4D). In the absence of anti-NKp46 mAb only a very weak killing of P815 was detected, with no clear subset difference (ranging from 16 to 100 lytic units [LU]), similar to previous findings [12] and fig. 5D.

### Effect of anti-CD2 mAbs on IFN-γ response from NK cells

The ability of CD2 cross-linking to trigger IFN-γ production from NK cells was assessed by culturing NK cells for 24 hours in plastic wells pre-coated with anti-CD2 mAbs, followed by measuring IFN-γ in the supernatants. In wells coated with one of the two tested anti-CD2-mAbs (mAb II, MUC2a), an IFN-γ response was measured, while in wells coated with the other (mAb I, BAQ95A), no response above background could be measured (Fig. 5A). This response occurred without rbIL-12, but was potentiated by the presence of this cytokine. The IFN-γ response by anti-NKp46 mAb was similar to previously obtained results [31], and was always higher than the anti-CD2 induced response. No IFN-γ above medium controls was measured with mAbs against CD8, which is present on most cultured NK cells [12], indicating that the response seen with MUC2A was CD2-specific.

### Effect of anti-CD2 mAbs on NK cytotoxicity

Available anti-bovine CD2 mAbs have not been previously tested for their ability to activate or inhibit NK cell cytotoxicity. To investigate this, bovine NK cells were pre-treated with the same two mAbs as mentioned above, and tested for interference with NK cytotoxicity in ^51^Cr release assays. Neither of these mAbs could alter the NK cytotoxicity against the bovine target cell lines BL-3.1 (Fig. 5B) or MDBK (Fig. 5C), while anti-NKp46 blocked most of the killing. A similar result was obtained with the target KN-31 using NK cells from two animals (not shown). No induction of redirected lysis was seen with the P815 target (Fig. 5D), while anti-NKp46 did, like previously reported [12].

## Discussion

The recent characterization of bovine NK cells [12] allows this important field of innate immunity to be studied in the cow. Human NK cells can be divided into functional subsets depending on the level of CD56 expression, but like in rats and mice, this antigen is probably not present on NK cells in the cow [11], thus not allowing a direct comparison with the human subsets. However, CD2 expression divides bovine NK cells into two distinct populations [12], and the scope of the present study was to examine CD2^+ ^and CD2^- ^bovine NK cell subsets for functional properties and status of activation.

Around 20% of NK cells in peripheral blood of cattle did not express CD2, similar to findings in man [20,21]. During two weeks of primary culture with IL-2, the NK cell population changed towards a dominance of the CD2^- ^phenotype. This change tended to be more pronounced in young animals, but was also present in adults. No intermediate forms were seen at any point, indicating that the two subsets were discretely preserved. However, under these unfractioned culturing conditions, the initial CD2^- ^population appeared to acquire a low density of CD2 receptors. In contrast, no such CD2 acquisition was seen when CD2^- ^NK cells were cultured separately. More detailed studies would be required to explain this phenomenon, but a conceivable hypothesis could be intercellular exchange of receptors during cell culture, as recently demonstrated [32]. We have consequently chosen to name these subsets CD2^-/low ^and CD2^+ ^when cultured in the presence of IL-2. IL-2 is reported to upregulate CD2 on human NK cells; partly as a minor intensity increase like observed here, but also as a proportional increase of the CD2^+ ^population [16,22], unlike our findings. Species differences may explain these conflicting proliferation patterns.

Interestingly, the CD2^- ^subset contained the highest relative numbers of IFN-γ^+ ^NK cells following cytokine stimulation, and IFN-γ secretion to the supernatant was also stronger following IL-2 stimulation. To our knowledge, experiments showing IFN-γ responses from corresponding subsets have not been reported in other species. Both subsets were cytotoxic against conventional target cells, the CD2^-/low ^most often, but not always, being the most effective killers. These findings are in agreement with reports from human NK cells where both subsets were comparably cytotoxic [22]. We conclude that both subsets had fully developed natural killer cell functions, the CD2^-/low ^NK cells being the more effective IFN-γ producers.

To identify appropriate activation markers in bovine NK cells we analyzed antigens known to be upregulated on other activated bovine lymphocytes [8,33]. We observed IL-2-induced upregulation of CD25, CD44 and CD45RO, in accordance with reports from human NK cells [34-37]. The unchanged CD11b expression and slight but transient CD11c upregulation in IL-2 culture was also similar to human NK cells [20].

In peripheral blood NK cells, two of the investigated markers (CD44 and CD25) were highest on CD2^- ^subset. Expression of CD25 on NK cells has been associated with higher proliferative potential [38], providing support for the increased proliferation of the CD2^- ^subset seen here. CD2, CD44 as well as the integrins are associated with cell adhesion, and notably, CD44 has been associated with binding to high endothelial venules [36]. Hence, the differential expression of these as well as CD11c on NK cell subsets could possibly relate to adhesive and/or migratory properties, although it has been warned against inferring *in vivo *migratory properties based on the expression of adhesion molecules alone [39].

In contrast to CD44 and CD25, CD45RO was moderately brighter on the CD2^+ ^subset. Increased expression of CD45RO has been reported to be a transient phenomena in human NK cells upon activation [37,40]. , but the role of this molecule in NK cells remains unclear [41,42]. It is noteworthy that in parallel to our findings with NK cells, bovine gamma delta (γδ) T-cells have been reported to express more CD44 and less CD45 in a CD2^- ^subset, although it was not stated which isoform of CD45 that was targeted [33]. Furthermore, human peripheral CD2^-/low ^T-cells were recently reported to show greater proliferative capacity as well as CD45RO expression, unlike CD2^+ ^T-cells; these were interpreted to be immature T-cells [43]. They were by definition CD3^+^, and therefore of a different lineage, but also in NK cells the lack of CD2 expression has been linked to immaturity [5,22]. The knowledge of NK cell development is still limited, but definition criteria for differentiation stages have recently been suggested [44]. According to those, the CD2^- ^subset presented here would qualify as mature NK cells since they show full cytotoxicity and IFN-γ production. The findings that they comprise up to 30% of circulating NK cells and express all NK receptors investigated on the mRNA level would appear paradoxical if they represent immature stages. Further studies would be required to target such aspects.

To complement these studies on NK cell subsets defined by CD2 expression, we examined the role of the CD2 molecule itself on bovine NK cells, by employing two bovine anti-CD2 mAbs in functional NK cell assays. Cross-linking by one of the mAbs induced a clear IFN-γ response, similar to studies in human NK cells [45]. Neither of the tested mAbs blocked cytotoxicity against NK-susceptible targets, and they did not induce redirected lysis of an FcR-bearing target. In human NK cells, significant blocking of cytotoxicity by CD2 mAbs usually does not occur [46], and no redirected lysis effect was observed [47], although cross-linking by conjugated mAbs against different epitopes could trigger cytotoxicity [48-50]. Similar observations have been done in mice [25]. The human CD2 molecule has later been shown to contain a continuum of overlapping epitopes, to which different mAbs may bind with various degrees of functional interference [51]. On this background, our experiments cannot exclude a CD2 function in cytotoxicity. Nevertheless, one CD2 mAb that apparently bound to a reactive epitope, since it could trigger IFN-γ production, did not affect target lysis. Together with the observation that CD2^- ^NK cells were fully cytotoxic and produced IFN-γ, we conclude that, similar to other species, the CD2 molecule is not necessary for bovine NK cell function.

Bovine NK or NK-like cells have sometimes been defined as CD3^-^/CD2^+ ^lymphocytes [7-9]. This may partly have been founded on reports that bovine PBMCs expressing CD2 were more cytotoxic than CD2^- ^PBMCs [10]. However, due to lack of NK-specific markers, those results could not be adjusted for the relative content of NK cells within each population. Our studies show that most NK cells in blood are CD2^+^. Considering that the majority of PBMCs do not express CD2, the proportion of CD2^- ^NK cells would constitute only a small fraction of this population, hence providing a likely explanation for the cytotoxicity pattern shown by Campos *et al*. [10]. Furthermore, in a report where CD3^-^/CD2^+ ^cells were classified as NK cells, CD3^-^/CD2^-^lymphocytes were reported as a not previously described "null cell" population [8]. Our present and previous studies [12] should provide evidence that these were most likely NK cells. In conclusion, we find that NK cell definitions based on a CD2^+ ^phenotype exclude an important subset of bovine NK cells.

## Conclusion

We have demonstrated here that CD2^- ^and CD2^+ ^subsets of bovine NK cells in peripheral blood have distinct phenotypic and functional properties, both subsets fulfilling criteria for being natural killer cells. The CD2^- ^subset of NK cells show phenotypical signs of being more activated, both obtained from blood and during the first days of IL-2 culture, they are better IFN-γ producers and have higher proliferative capacity. Both subsets are cytotoxic and carry mRNA transcripts for a set of common NK cell receptors. One anti-CD2-mAb could incite an IFN-γ response from bovine NK cells, but no alteration of cytotoxicity was found by two tested mAbs. The results presented here establish NKp46^+^/CD2^- ^cells as a distinct, activated and fully functional subset of NK cells in the cow, with a particular capacity for IFN-γ production.

## Methods

### Isolation and culture of bovine NK cells and subsets

Clinically healthy Norwegian Red dairy cattle of both sexes and of different ages above 3 months were used in this study. Blood samples were collected in tubes with EDTA as anticoagulant. Peripheral blood mononuclear cells (PBMC) were obtained by density gradient centrifugation using Lymphoprep (Axis-Shield, Oslo, Norway). NK cells were isolated and cultured as previously described [12]. Briefly, PBMC were positively selected using mAb against NKp46 (CD335) (AKS1;IgG1; Serotec, Oxford, UK) and immunomagnetic anti-mouse pan IgG beads (Dynal, Oslo, Norway), and cultured in RPMI medium supplemented with 60μg/ml penicillin, 100 μg/ml streptomycin, 1 mM sodium pyruvate, non-essential amino acids, 50μM 2- mercaptoethanol (all GIBCO, Invitrogen) with 10% FCS and 100 U/ml rbIL-2, produced as described below. After 24–48 h, beads had undergone spontaneous detachment and were magnetically removed. Selection of NK cell subsets was performed after 24 h, by applying the anti-CD2 mAb MUC2A (VMRD, Pullman, WA, USA) and MACS anti-mouse IgG MicroBeads and LS columns (both Miltenyi Biotech, Bergish Gladbach, Germany) according to the manufacturer's instructions. To enhance purity, positive selection was repeated using a new LS column, and depletion repeated using an LD column. NK cells or subsets were cultured further for the appropriate length of time in rbIL-2-containing medium.

### Flow cytometry

Two- or three-coloured flow cytometric analysis of surface receptors was performed on whole blood samples or cultured cells, as previously described [12], using the following antibodies: CD2 (MUC2A;IgG2a, BAQ95A;IgG1, or B26A4;IgM), CD11b (BAQ147A;IgM), CD11c (BAQ153A;IgM), CD25 (CACT108A;IgG2a), CD44 (BAG40A;IgG3), CD45RO (GC44A;IgG3) (all from VMRD), and NKp46 (AKS1). Subtype-specific secondary antibodies were conjugated with FITC or PE (Southern Biotech, Birmingham, AL, USA), or with Alexa Fluor 488 or Alexa Fluor 633 (Molecular Probes, Invitrogen). The samples were analyzed on a FACS CALIBUR flow cytometer (Becton Dickinson), equipped with Cell-Quest Pro software. 5 × 10^4 ^viable cells as gated in the forward side scatter plot were analyzed.

### CFSE proliferation analysis

For cell proliferation studies, positively selected NK cells cultured for 20 h in the presence of rbIL-2 were incubated with 5 μM CFSE in PBS with 0.2% BSA for 10 min followed by addition of 5 ml cold RPMI and incubation on ice for 5 min. The cells were washed three times in RPMI and cultured in the presence of 100 U/ml rbIL-2 as described above. To obtain non-proliferating NK cells as controls, rbIL-2 was added at 1U/ml to washed NK cells; this amount was obtained by titrating rbIL-2 to an amount that kept the cells viable, but non-proliferating, as measured by cell counting and incorporation of ^3^H labeled thymidine.

### Detection of intracellular and secreted IFN-γ

Bovine PBMCs were analyzed for IFN-γ producing cells as described previously [52], with modifications. Briefly, heparinized whole blood was incubated overnight in the absence or presence of rbIL-2 (200 U/ml), or rbIL-12 (5 U/ml) (kindly provided by Jayne Hope, Institute of Animal Health, Compton, UK). Twelve hours before harvesting, Brefeldin A (10 μg/ml) (Sigma) was added. PBMC were isolated by density gradient centrifugation and surface labelling was performed with mAbs against NKp46 (AKS1) and CD2 (MUC2A), and following permeabilization and fixation the anti-bovine IFN-γ mAb clone 6.6 (IgG2b) was applied (kindly provided by Gregers Jungersen, Danish Institute for Food and Veterinary Research, Copenhagen). 10^5 ^cells per sample were analyzed in a flow cytometer as described above.

For assessment of secreted IFN-γ, NK cell subsets pre-cultured for one week in the presence of rbIL-2 were incubated for 24 h in triplicates of 10^5^, in the presence or absence of 0.5 U/ml rbIL-12. To assess the ability of mAb-ligation of the CD2 receptor to trigger IFN-γ production, NK cells were cultured in triplicates of 2 × 10^5 ^in 96-well MaxiSorp plates (NUNC, Denmark) pre-coated overnight in 0.05 M carbonate buffer (pH 9.6) with either of two anti-CD2 mAbs (MUC2A, BAQ95A), with anti-NKp46 mAb (AKS1), anti-CD8 mAb (BAQ111A;IgM) or none. In both types of experiment, medium was always containing 100U/ml rbIL-2. IFN-γ production in the supernatants was assessed using an ELISA (Bovine IFN-γ EASIA, Biosource, Nivelles, Belgium) according to the manufacturer's instructions. Concentrations were calculated from a standard curve made using purified bovine IFN-γ, kindly provided by Dr Stephen Jones (Pfizer, Melbourne, Australia).

### NK cell cytotoxicity assays

The cytotoxic capacity of IL-2 activated bovine NK cells or subsets was tested in a standard ^51^Cr release assay as previously described [12], using the bovine target cell lines MDBK, BL3.1 or KN-31 (generic calf kidney cell line) or murine P815. The BL3.1 line is MHC class I-impaired, thus potentially devoid of important NK-cell inhibitory ligands [53], while P815 expresses Fcγ-receptors, allowing reverse antibody linking for redirected lysis. Specific ^51^Cr release was calculated on the basis of the ratio [(sample release – spontaneous release)/(total release – spontaneous release)] and lytic units (LU) were calculated as the capacity to kill 20% of targets contained within 10^7 ^effectors, as described by Bryant and colleagues [54]. In redirected lysis assays, the effector cells were preincubated for 20–30 min with 1 μg/ml of mAbs against NKp46 (AKS1), CD8 (CACT80C) or CD2 (BAQ95A) (all IgG1).

### Expression of recombinant bovine interleukin-2

As bovine NK cells do not proliferate in response to human IL-2 [11,12], bovine rbIL-2 was produced as follows: mRNA was isolated from conA stimulated bovine PBMC with Dynabeads Oligo (dT)_25 _(Dynal) and cDNA was synthesized by SuperScript™ II RNase H^- ^Reverse Transcriptase using oligo (dT)_12–18 _(both Invitrogen) The coding region of bovine IL-2 [GenBank: M12791] [55] was amplified by PCR using *PfuUltra*™ High-Fidelity DNA Polymerase (Stratagene, La Jolla, CA) and gene-specific primers with BamHI and XhoI restriction sites: 5' GGATCCTCAACTCCTGCCACAATGTA 3' and 5' CTCGAGTTAAGACTAACAGTTACAAAAGGT 3'. The PCR product was purified by agarose gel electrophoresis and QIAquick Gel Extraction Kit (Qiagen, Hilden, Germany), incubated with *Taq *DNA polymerase (Invitrogen), cloned into pCR^® ^2.1-TOPO Vector (Invitrogen), released by BamHI and XhoI digestion and subcloned into pcDNA I (Invitrogen). The correctness of this construct was verified by sequencing. 20 μg of plasmid DNA was mixed with 120 μl Lipofectamine (Invitrogen) in 2.8 ml Opti-MEM (Invitrogen) and incubated for 20–30 min at room temperature. 11.2 ml Opti-MEM were then added and the mixture transferred to a 160-cm^2 ^flask containing a 70% confluent layer of 293T cells that had been washed three times with Opti-MEM. After 6 h, 14 ml of RPMI 1640 medium/20% FCS (Invitrogen) was added. After another 6 h, cells were washed twice in HBSS (Invitrogen) and RPMI/10 % FCS was added. The medium, containing rbIL-2, was harvested twice within three days, sterile filtered and assayed in an IL-2 bioassay based on proliferation of 4 days ConA stimulated PBMC as measured by ^3^H labeled thymidine incorporation. 1 U is defined as the amount of IL-2 inducing half the maximal proliferation of ConA blasts. The supernatant contained 10000 U rbIL-2/ml, was stored at -20°C, and was used without further purification at the described concentrations.

### Detection of gene expression

The expression of several NK cell receptors was detected in CD2^+ ^and CD2^- ^NK cell subsets by reverse transcriptase PCR. mRNA was harvested by μMACS mRNA Isolation Kit (Miltenyi Biotech) from 0.5–1 × 10^7^cells and cDNA was synthesized by SuperScript™ III RNase H^- ^Reverse Transcriptase using oligo (dT)_12–18. _The amount of cDNA template was measured using NanoDrop spectrophotometer (NanoDrop, Wilmington, DE, USA). Intron spanning primers generating products of about 500 bp were designed based on sequences previously described [29,30]. NKp46 forward: 5' GAGCGATGCCTTCCACACTTGC 3' and reverse 5' GGTTGTTGTAGGAGCCAAAGCATCTGT 3'; annealing temperature 68°C, CD16: forward 5' GTGGTGGCACAATGGGAC 3' and reverse 5' GCCATCCCTCCATTCCTC 3', annealing temp. 62°C; KIR: forward 5' AATGTGACCCTCCGCTGTC 3' and reverse 5' AACAGTGGGTCGCTGGAGT 3', annealing temp. 64°C; CD94/KLRD1: forward 5' CCTTGTGTTGATGGCTGCTT 3' and reverse 5' AAAATCCTTCCTCTTGGGTT 3' annealing temp. 58°C; KLRJ forward 5' TGTGGATTCTTTGGTTACCAG 3' and reverse 5' TGAGGCCAGAAGGAAGAGAA 3' annealing temp. 59°C; GAPDH forward AGTGGGGTGATGCTGGTGCT, reverse TCCAGGCGGCAGGTCA, annealing temp. 66°C (477bp product). 600 ng of cDNA was added to three parallel PCR reactions run at 20, 25 or 30 cycles with Dynazyme (Finnzymes, Espoo, Finland). Initial analysis revealed that NKp46 was equally expressed in both NK cell subsets in comparision to GAPDH (not shown), and its surface expression was also at equal levels. Intensity of PCR products was determined using NKp46 as a reference gene, using KODAK 1D Image Analysis software (Eastman Kodak, New Haven, CT, USA).

## Authors' contributions

PB designed the study and carried out cell isolation, separation and culture, flow cytometry, cytotoxicity assays, participated in IFN-γ assays, CFSE and gene expression analysis, performed literature searches and drafted the manuscript. IO and SK carried out CFSE proliferation analysis and intracellular IFN-γ assays. IB carried out rbIL-2 expression and gene expression analyses. GMJ carried out gene expression analyses, IFN-γ ELISAs and participated in cell isolation and culture. AKS conceived, coordinated and supervised the study, and participated in all experiments as well as manuscript preparation.
